# The *β*_4_-Subunit of the Large-Conductance Potassium Ion Channel K_Ca_1.1 Regulates Outflow Facility in Mice

**DOI:** 10.1167/iovs.61.3.41

**Published:** 2020-03-23

**Authors:** Jacques A. Bertrand, Martin Schicht, W. Daniel Stamer, David Baker, Joseph M. Sherwood, Elke Lütjen-Drecoll, David L. Selwood, Darryl R. Overby

**Affiliations:** 1 Department of Bioengineering, Imperial College London, London, United Kingdom; 2 Department of Anatomy II, University of Erlangen-Nürnberg, Erlangen, Germany; 3 Department of Ophthalmology, Duke University, Durham, North Carolina, United States; 4 Department of Neuroinflammation, University College London Institute of Neurology, University College London, London, United Kingdom; 5 Department of Medicinal Chemistry, Wolfson Institute for Biomedical Research, University College London, London, United Kingdom

**Keywords:** trabecular meshwork, outflow facility, ion channels, mechanotransduction, mouse models

## Abstract

**Purpose:**

The large-conductance calcium-activated potassium channel K_Ca_1.1 (BK_Ca_, maxi-K) influences aqueous humor outflow facility, but the contribution of auxiliary *β*-subunits to K_Ca_1.1 activity in the outflow pathway is unknown.

**Methods:**

Using quantitative polymerase chain reaction, we measured expression of *β*-subunit genes in anterior segments of C57BL/6J mice (*Kcnmb1-4*) and in cultured human trabecular meshwork (TM) and Schlemm's canal (SC) cells (*KCNMB1-4*). We also measured expression of *Kcnma1*/*KCNMA1* that encodes the pore-forming *α*-subunit. Using confocal immunofluorescence, we visualized the distribution of *β*_4_ in the conventional outflow pathway of mice. Using iPerfusion, we measured outflow facility in enucleated mouse eyes in response to 100 or 500 nM iberiotoxin (IbTX; *N* = 9) or 100 nM martentoxin (MarTX; *N* = 12). MarTX selectively blocks *β*_4_-containing K_Ca_1.1 channels, whereas IbTX blocks K_Ca_1.1 channels that lack *β*_4_.

**Results:**

*Kcnmb4* was the most highly expressed *β*-subunit in mouse conventional outflow tissues, expressed at a level comparable to *Kcnma1*. *β*_4_ was present within the juxtacanalicular TM, appearing to label cellular processes connecting to SC cells. Accordingly, *KCNMB4* was the most highly expressed *β*-subunit in human TM cells, and the sole *β*-subunit in human SC cells. To dissect functional contribution, MarTX decreased outflow facility by 35% (27%, 42%; mean, 95% confidence interval) relative to vehicle-treated contralateral eyes, whereas IbTX reduced outflow facility by 16% (6%, 25%).

**Conclusions:**

The *β*_4_-subunit regulates K_Ca_1.1 activity in the conventional outflow pathway, significantly influencing outflow function. Targeting *β*_4_-containing K_Ca_1.1 channels may be a promising approach to lower intraocular pressure to treat glaucoma.

Intraocular pressure (IOP) is determined by aqueous humor outflow facility. With decreasing facility, IOP becomes elevated, and elevated IOP is the major risk factor for glaucoma. The trabecular meshwork (TM) and inner wall endothelium of Schlemm's canal (SC) regulate outflow facility.^1^^,^^2^ Although the precise mechanism of facility regulation is not fully understood,^3^ volume, stiffness, and contractility of TM and SC cells are known to play important roles.^4^^–^^8^

The large-conductance calcium-activated potassium channel K_Ca_1.1 (BK_Ca_, maxi-K or Slo1) regulates cell volume and contractility in smooth muscle cells,^9^ as well as in TM and SC cells.^5^^,^^10^^–^^12^ The K_Ca_1.1 channel opener, NS1619, increases outflow facility in porcine anterior segments.^13^ NS1619 also inhibits the decrease in outflow facility following perfusion with hypotonic saline in bovine anterior segments^5^ and, likewise, decreases TM cell volume^13^ and smooth muscle cell contractility.^14^ Conversely, blocking K_Ca_1.1 with iberiotoxin (IbTX) potentiates the facility decrease in response to hypotonic saline^5^ and inhibits the cell volume decrease in response to NS1619.^13^ IbTX also inhibits the facility increasing effect of nitric oxide donors in porcine anterior segments.^15^ K_Ca_1.1 is thus involved in outflow facility regulation, apparently by regulating the volume or contractility of SC or TM cells.

K_Ca_1.1 is composed of four *α*-subunits that form the potassium-selective transmembrane pore. Membrane depolarization or increased cytosolic calcium may activate K_Ca_1.1, leading to channel opening, potassium efflux, and membrane depolarization.^16^ Association between *α* and auxiliary *β*-subunits, of which there are four types (*β*_1-4_),^17^ regulates K_Ca_1.1 sensitivity to voltage and calcium. *β*-subunits are differentially expressed in a tissue-specific manner and affect the pharmacology of K_Ca_1.1. For example, *β*_4_ renders K_Ca_1.1 relatively resistant to IbTX but sensitive to martentoxin (MarTX), which has a smaller effect on K_Ca_1.1 channels lacking *β*_4_.^18^^–^^20^

In this study, we examine the expression of genes encoding the *β*-subunits of K_Ca_1.1 in mouse anterior segments *(Kcnmb1-4*) and in human TM and SC cells *(KCNMB1-4*). We focus on *β*_4_, examining its localization in the TM and inner wall endothelium of SC. Using MarTX, we examine the influence of *β*_4_ on outflow facility in enucleated mouse eyes. We also examine the effect of IbTX to investigate the influence of K_Ca_1.1 channels that lack *β*_4_ on outflow facility.

## Methods

### Gene Expression

We used quantitative polymerase chain reaction (qPCR) to measure the expression of *Kcnma1* and *Kcnmb1-4* in mouse anterior eye segments and *KCNMA1* and *KCNMB1-4* in cultured human TM and SC cells. For mouse anterior segments, C57BL/6J mice (*N* = 3, 13-week-old males, Charles River UK Ltd., Margate, UK) were euthanized by cervical dislocation. Eyes were enucleated, trimmed of extraocular tissue, and hemisected at the equator. The lens was removed, and the anterior segments homogenized in TRIzol using a rotor-stat tissue homogenizer (Ultra-Turrax T10; VWR, Leicestershire, UK). Total RNA was extracted using PureLink RNA spin columns following manufacturer protocols (ThermoFisher Scientific, Waltham, MA, USA). RNA content was measured using a spectrophotometer (NanoDrop ND-1000; ThermoFisher Scientific), and 2 µg of RNA was used to synthesize cDNA by reverse transcription (Superscript VILO; ThermoFisher Scientific). qPCR was carried out using TaqMan master mix and primers (see Supplementary Table S1). *GAPDH/Gapdh* was used as the reference gene. cDNA was analyzed in triplicate (QuantStudio 6 Flex; Applied Biosystems, ThermoFisher Scientific). Expression level relative to *GAPDH/Gapdh* was calculated using the ΔCt method. Homogenized mouse brain tissue, which expresses *Kcnma1* and *Kcnmb4*,^21^ was used as a positive control. Mouse 3T3-L1 fibroblasts (passage 25), which do not express K_Ca_1.1 channels,^22^ were used as a negative control. As the anterior segment contains various tissues, this approach was unable to attribute expression to TM or SC directly. Further, pooling tissues together may mask the actual gene expression within the TM or SC.

Human TM and SC cells were isolated and characterized from human donor eyes following established techniques.^23^^–^^26^ Cells were grown to confluency in T25 flasks and lysed using TRIzol. RNA extraction and quantification followed the methods described earlier for mouse anterior segments. These studies used TM cell strains TM86, TM93, and TM134 from donors aged 3 months, 35, and 51 years, respectively, and SC cell strains SC56 and SC67 from donors aged 29 and 44 years, respectively. TM and SC cells were used between passage 4 and 6. EA.hy926 human endothelial cells (ATCC CRL-2922; LGC standards, Middlesex, UK) were used at passage 8 as a positive control for *KCNMA1* and *KCNMB4* expression.^27^

### Microscopy

Whole globes from three adult mice (C57BL/6J) were fixed in 4% paraformaldehyde (Roth, Karlsruhe, Germany) for 4 hours and washed in PBS. The eyes were hemisected at the equator, and 10 µm sagittal cryostat sections were cut. The sections were incubated in BLOTTO´s Blocking Buffer (ThermoFisher Scientific) at room temperature for 1 hour to reduce nonspecific staining. Following three washes in Tris-buffered PBS, specimens were incubated with the primary antibody (Table 1) at 4°C overnight, washed three times with PBS, and incubated with a secondary antibody (goat anti-rabbit Alexa 488; Invitrogen A11070; 1:1000) for 75 minutes. For double immunolabeling, specimens were washed three times with PBS and incubated with the second primary antibody (Table 1) at 4°C overnight. After washing three times with PBS, sections were incubated with secondary antibody (goat anti-rat Cy3; Dianova, Dianova GmbH, Hamburg, Germany 112-169-003; 1:1000) for 1 hour. Sections were mounted on glass slides with a 1:1 mixture of PBS and glycerol containing DAPI to label cell nuclei (10 µL of 2 mg/mL). The slides were examined with a Keyence Biorevo BZ9000 microscope (Keyence, Neu-Isenburg, Germany).

**Table 1.** Antibodies Used for Immunofluorescence Microscopy

Protein	Host Species	Company, Catalog Number	Dilution in PBS
Anti-K_Ca_1.1 β_4_	Rabbit	Alomone Labs; APC-061	1:50
PECAM-1/CD31	Rat	Biolegend; 102401; Clone C390	1:50

For electron microscopy, one MarTX perfused mouse eye (100 nM) and its paired contralateral vehicle perfused eye, were postfixed in OsO_4_ and dehydrated. Whole eyes were embedded in Epon resin and 1 µm semithin sagittal sections were cut using an ultramicrotome (Ultracut E; Reichert Jung, Vienna, Austria). Semithin sections were stained with toluidine blue and examined with a Keyence Biorevo BZ9000 microscope. Ultrathin sections through the TM were then cut, stained with uranyl acetate and lead citrate, and viewed with a transmission electron microscope (JEM 1400 plus; JEOL, MA, USA). Four regions were examined per eye.

### Outflow Facility Measurements

We measured the effect of IbTX and MarTX on outflow facility in enucleated eyes from C57BL/6J mice (13-week-old males; Charles River UK Ltd., Margate, UK). We used a paired experimental design, in which one eye was perfused with IbTX or MarTX and the contralateral eye perfused with vehicle. We measured the relative difference in outflow facility between treated and untreated paired eyes. All procedures on living mice were carried out under the authority of a United Kingdom Home Office project license and adhered to the ARVO Statement for the Use of Animals in Ophthalmic and Vision Research.

IbTX was purchased from Tocris Bioscience (Abingdon, UK). MarTX was custom synthesized by Peptide Protein Research Limited (Fareham, UK) with the sequence FGLIDVKCFASSECWTACKKVTGSGQGKCQNNQCRCY, as determined by Ji et al.^28^ This sequence contains the N-terminal phenylalanine, which may be lacking from some commercial suppliers, but is critical for MarTX function.^29^ MarTX was modified by the addition of propargyl (Pra) to the lysine at position 7 with the sequence FGLIDV[Pra]CFASSECWTACKKVTGSGQGKCQNNQCRCY. Pra labeling was chosen because it is compatible with click-chemistry, and thereby allows fluorescent click labeling to localize MarTX in fixed specimens. Separate studies revealed successful MarTX labeling in fixed EA.hy926 cells, but labeling was undetectable within the mouse TM in situ (data not shown).

For perfusions, mice were humanely culled by cervical dislocation. Eyes were enucleated, affixed to a support platform using tissue glue, and submerged in PBS at 35°C. Using a micromanipulator and dissection microscope, the anterior chamber was cannulated within 10 to 15 minutes of death using a 33-gauge beveled metal needle (NanoFil, NF33BV-2; World Precision Instruments, Sarasota, FL, USA) connected to the iPerfusion system (Bioengineering Department, Imperial College London, UK).^30^ The vehicle was Dulbecco's PBS containing divalent cations and 5.5 mM glucose (collectively referred to as DBG) that was passed through a 0.2-µm filter prior to use. Paired eyes were perfused with either DBG or DBG containing IbTX or MarTX. We examined the effect of 100 nM IbTX (*N* = 4 pairs), 500 nM IbTX (*N* = 5), and 100 nM MarTX (*N* = 12).

IOP was set to 9 mm Hg for 1 hour to pressurize and acclimatize the eye to the perfusion environment. Flow into the eye was then measured over 7 increasing pressure steps from 6.5 to 16.5 mm Hg. The flow rate at each step was considered to have reached stability when the ratio of the flow rate to pressure changed by less than 0.1 nL/min/mm Hg per minute over a 5-minute window. Three pressure steps from three perfusions failed to reach stability and were excluded from further analysis. The stable flow rate *Q* and stable pressure *P* were calculated over the last 4 minutes of each step, and the *Q* − *P* data were fit by a power-law relationship of the form

(1) $$Q=C_{r}{\left(\frac{P}{P_{r}}\right)}^{b}P$$

The fitting yields *C_r_*, which represents the value of outflow facility at a reference pressure *P_r_* of 8 mm Hg, and *b* that characterizes the nonlinearity of the *Q*-*P* relationship.^30^ We then calculated the relative difference in *C_r_* between treated and untreated contralateral eyes, defined as the ratio of *C_r_* in the treated eye relative to that in the contralateral control eye minus unity. We tested whether the relative difference in facility was statistically different from zero using a weighted *t*-test on the log-transformed data, as previously described.^30^ Facility values and relative changes in facility were then converted back into the linear domain and reported in terms of the geometric mean and the 95% confidence interval on the mean.

## Results

### Gene Expression

By qPCR, we detected positive expression (Ct < 35) of *Kcnma1*, *Kcnmb1*, and *Kcnmb4* in mouse anterior segments (*N* = 3; Table 2 and Supplementary Fig. S1A). *Kcnmb2* and *Kcnmb3* had Ct values higher than 35 and 40, respectively, and were thus considered low expression or below the limit of quantification. ΔCt values (∆Ct = Ct[gene] – Ct [*Gapdh*]) for *Kcnma1* and *Kcnmb4* were similar for mouse anterior segments (7.6 and 8.5, respectively), whereas ∆Ct for *Kcnmb1* was greater (10.1), indicating roughly four-fold lower expression (Supplementary Fig. S1A). Homogenized mouse brain was positive for all genes except *Kcnmb3*, which was expressed at low levels. Mouse 3T3-L1 fibroblasts expressed low levels of *Kcnma1* and *Kcnmb4. Kcnmb1-3* were below the limit of quantification (40 cycles).

**Table 2.** Expression of Genes Encoding the K_Ca_1.1 Channel *α-*Subunit and the Four *β*-Subunits in Mouse Tissues (*N* = 3 mice) and Human Cells Measured by qPCR

	Gene Expression				
	*Kcnma1*	*Kcnmb1*	*Kcnmb2*	*Kcnmb3*	*Kcnmb4*
**Mouse Tissue**				
Brain	+	+	+	+/−	+
Anterior Segment	+	+	+/−	−	+
**Human Cells**	*KCNMA1*	*KCNMB1*	*KCNMB2*	*KCNMB3*	*KCNMB4*
Ea.Hy926	+	+/−	−	−	+
hSC58	+	−	−	−	+
hSC67	+	−	−	−	+
hTM93	+	+	+/−	−	+
hTM86	+	+/−	+/−	−	+
hTM134	+	+	+/−	−	+

Positive (+) indicates all samples tested gave Ct values lower than 35 cycles. Positive/negative (+/−) indicates that at least one sample tested gave a Ct value between 35 and 40 cycles. If all samples tested gave Ct values greater than 40 cycles, they were considered indistinguishable from background and marked negative (−). Full details of Ct values are available in Supplementary Figure S1.

Human TM cells expressed *KCNMA1*, *KCNMB1*, and *KCNMB4*, but low levels of *KCNMB2* and undetectable levels of *KCNMB3* (Table 2 and Supplementary Fig. S1B). Human SC cells expressed *KCNMA1* and *KCNMB4*, but no other *β*-subunit genes (Table 2 and Supplementary Fig. S1B). *β*_4_ was the most highly expressed *β*-subunit in TM cells, and the sole *β*-subunit in SC cells. EA.hy926 cells, used as a positive control, primarily expressed *KCNMA1* and *KCNMB4*. TM and SC cells express similar amounts of *KNCMA1* relative to EA.hy926 cells, but several-fold higher levels of *KCNMB4* (Supplementary Fig. S1B).

We then analyzed published microarray data (GEO ID: GSE32169) from primary human TM cells deposited by Porter et al.^31^ Consistent with our qPCR data described earlier, *KCNMB4* was the most highly expressed *β*-subunit gene, with expression levels comparable to *KCNMA1*. *KCNMB1* and *KCNMB3* were expressed at lower levels, and *KCNMB2* was the lowest expressed (Supplementary Fig. S1C). These data show that *β*_4_ is the most highly expressed auxiliary *β*-subunit of the K_Ca_1.1 channel in mouse anterior segments, as well as in cultured human TM and SC cells.

### Immunofluorescence Localization

**Figure 1.**
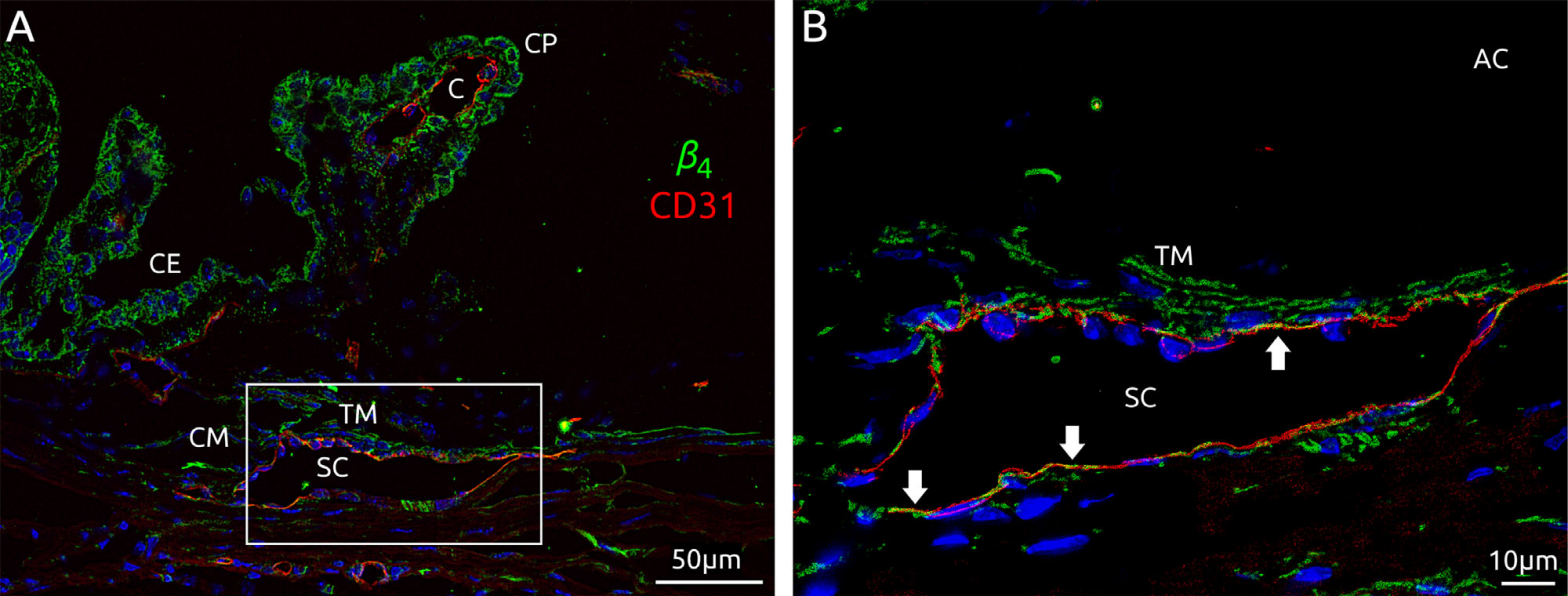
Localization of *β*_4_ in the iridocorneal angle of the mouse eye. Boxed region in (**A**) is shown magnified in (**B**). Labeling of *β*_4_ is shown in *green*, CD31 in *red*, and nuclei stained by DAPI in *blue*. AC, anterior chamber; CM, ciliary muscle; CP, ciliary process; CE, ciliary epithelium; C, ciliary process capillary. *Arrows* show areas of colocalization of *β*_4_ with CD31 along the inner and outer walls of SC.

We then examined the localization of *β*_4_ within the conventional outflow pathway of C57BL/6J mice using confocal immunofluorescence (Fig. 1). *β*_4_ was observed in the TM, labeling subendothelial cells underlying the inner and outer walls of SC. Continuous bands of *β*_4_ labeling were observed in the internal TM, suggestive of labeling along trabecular beams. Punctate labeling of *β*_4_ was observed along the inner and outer walls of SC, identified by CD31. The punctate *β*_4_ labeling along SC endothelium often appeared as extensions of *β*_4_ labeling within the subendothelial space, suggestive of junctional processes connecting subendothelial or juxtacanalicular cells to the basal surface of SC endothelium.^32^^–^^34^ *β*_4_ labeling was also observed along ciliary process capillaries, the ciliary epithelium, and in nerve bundles within the sclera and cornea. Moderate *β*_4_ labeling was observed in the ciliary muscle.

### Outflow Facility

**Figure 2.**
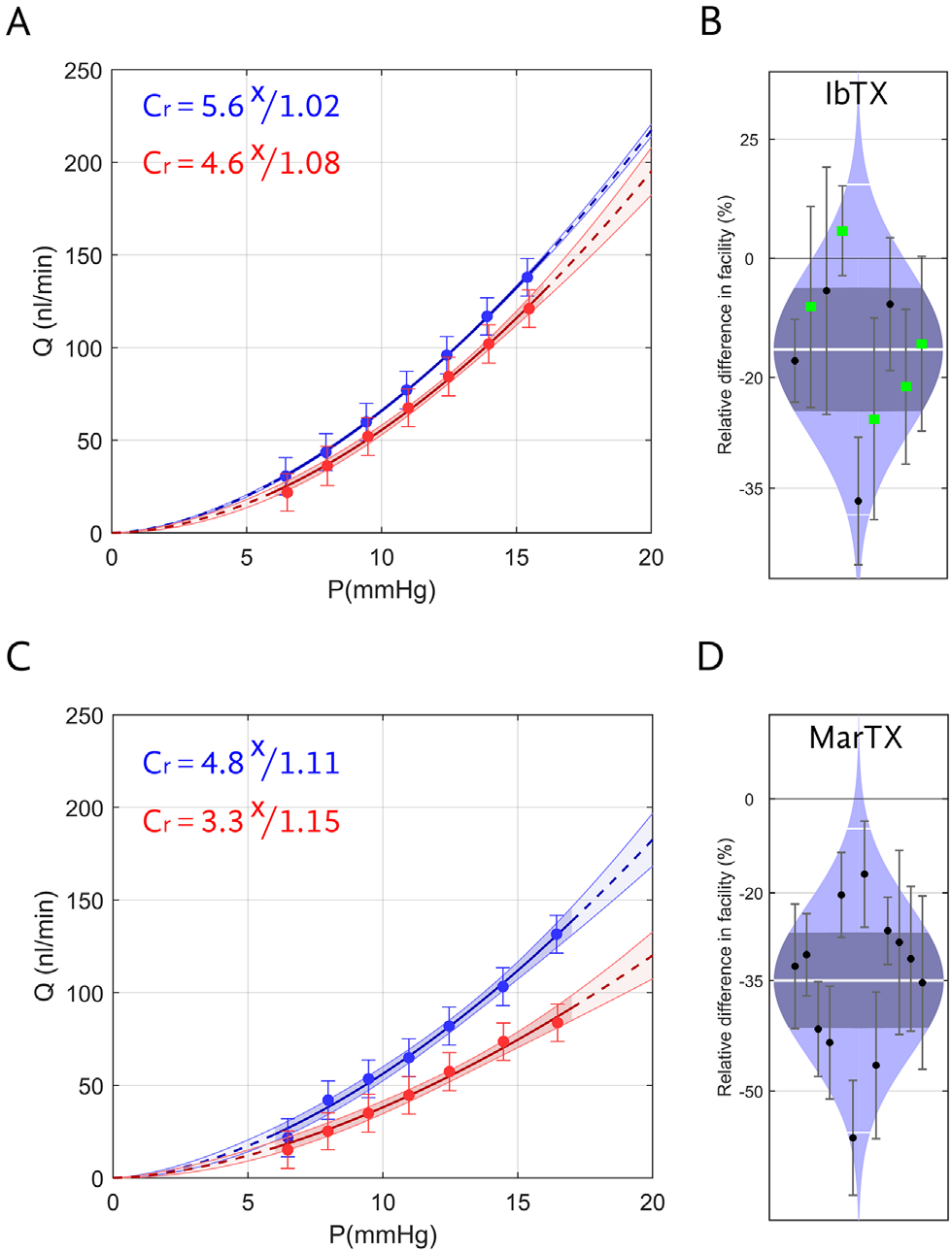
Representative flow-pressure (Q-P) plots of eyes treated with IbTX (**A**) or MarTX (**C**) versus vehicle-perfused contralateral eyes. *Error bars* are 95% confidence intervals. Relative difference in facility for IbTX (**B**) and MarTX (**D**) treated eyes. The relative difference in facility is defined as the ratio of *C_r_* in the treated eye relative to that in the untreated contralateral eye minus unity, expressed as a percentage. Each data point represents the relative difference in facility for an individual mouse. *Error bars* are 95% confidence intervals. Shaded regions represent the best estimate of the sample distributions, with the central *white line* representing the geometric mean. Dark central bands represent the 95% confidence interval on the mean, and the outer *white lines* represent the limits encompassing 95% of the population. *Green* squares in (*B*) correspond to data obtained from 500 nM IbTX and *black circles* from 100 nM. The *C_r_* values appearing in (**A**, **C**) correspond to the Q-P data shown in each plot, in units of nL/min/mm Hg.

We then examined the effect of two K_Ca_1.1 blockers, IbTX and MarTX, on outflow facility in enucleated mouse eyes using the iPerfusion system.^30^ IbTX predominately blocks K_Ca_1.1 channels that contain only *α*-subunits or *α*+*β*_1_ subunits, but IbTX does not effectively block K_Ca_1.1 that contain *β*_4_.^18^ In response to 100 or 500 nM IbTX, outflow facility decreased by 16% [6%, 25%] (geometric mean, [95% confidence interval]) relative to contralateral eyes that were perfused with vehicle (P = 0.01, n = 9 pairs; paired two-tailed t-test; Figs. 2A, 2B). Data for both concentrations were pooled together because there was no statistical difference between 100 and 500 nM IbTX (*P* = 0.6; *n* = 4 and 5) for the present data (unpaired two-tailed *t*-test). *C_r_* for IbTX-treated eyes was 5.0 [3.8, 6.2] nL/min/mm Hg versus 5.9 [4.8, 7.1] nL/min/mm Hg for vehicle-treated eyes.

MarTX is a potent and selective blocker of *β*_4_-containing K_Ca_1.1 channels.^19^ In response to 100 nM MarTX, outflow facility decreased by 35% [27%, 42%] (*P* < 0.0001, *n* = 12; Figs. 2C, 2D) relative to vehicle-treated contralateral eyes. *C_r_* for MarTX-treated eyes was 4.3 [3.2, 5.5] nL/min/mm Hg compared with 6.7 [5.5, 8.0] nL/min/mm Hg for vehicle-treated eyes. The effect of MarTX on *C_r_* was significantly greater than that of IbTX (*P* = 0.01, unpaired two-tailed *t*-test).

**Figure 3.**
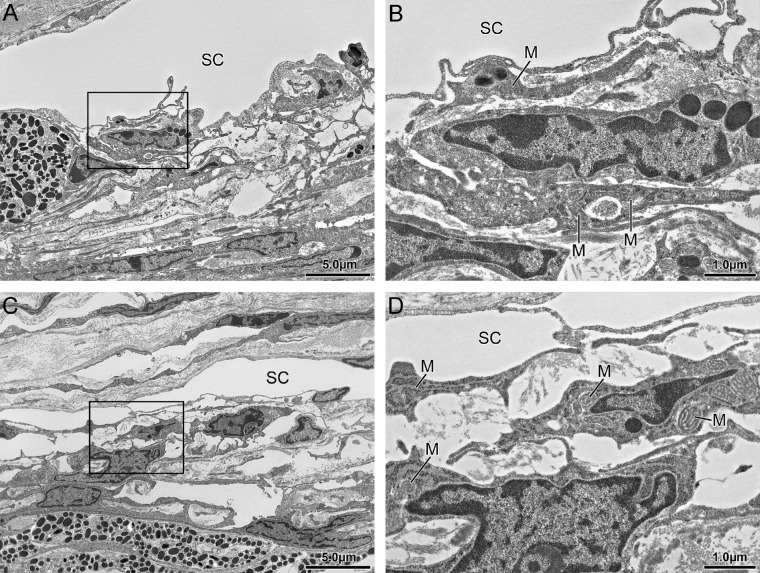
Sagittal sections of paired mouse eyes perfused with vehicle (**A**, **B**) or 100 nM MarTX (**C**, **D**) imaged by transmission electron microscopy. Boxed regions in (**A**, **C**) are shown magnified in (**B**, **D**). M, mitochondria. *Scale bars* are 5 µm in (**A**, **C**) and 1 µm in (**B**, **D**).

To examine whether MarTX induces cellular toxicity in the outflow pathway, we used transmission electron microscopy to visualize the ultrastructure of the TM in a pair of eyes perfused with MarTX or vehicle. There were no obvious morphological differences between MarTX and vehicle-perfused eyes. In both cases, the inner wall of SC appeared continuous, and the juxtacanalicular connective tissue (JCT) contained loose extracellular matrix interspersed by JCT cells that extended connections to the inner wall and TM beams (Figs. 3A, 3C). The mitochondria appeared normal without any evidence of swelling that would indicate toxicity, and cell membranes and cell–cell connections appeared intact (Figs. 3B, 3D). This suggests that MarTX does not induce an overtly toxic effect in the outflow pathway.

## Discussion

The large-conductance calcium-activated potassium channel K_Ca_1.1 is a known regulator of outflow facility. Opening K_Ca_1.1 with NS1619 increases outflow facility in porcine anterior segments.^13^ Conversely, blocking K_Ca_1.1 with IbTX prevents the increase in outflow facility following treatment with nitric oxide^15^ or perfusion with hypotonic saline.^5^ Here we show that blocking K_Ca_1.1, with either IbTX or MarTX, independent of nitric oxide or any other stimulus, decreases outflow facility in enucleated mouse eyes. Thus across different species, opening the K_Ca_1.1 channel coincides with increasing outflow facility, whereas blockade of the K_Ca_1.1 channel corresponds with decreasing outflow facility.

K_Ca_1.1 may modulate outflow facility by influencing cell contractility. Opening K_Ca_1.1 leads to potassium efflux and membrane hyperpolarization that inactivates voltage-gated calcium channels.^16^ The associated reduction in cytosolic calcium leads to relaxation in smooth muscle cells.^9^ Similarly, opening K_Ca_1.1 leads to relaxation in TM and ciliary muscle cells and a reduction in calcium-induced actin polymerization.^7^^,^^35^ Cell relaxation is typically associated with increasing outflow facility, as occurs following treatment with rho kinase inhibitors^36^^,^^37^ or nitric oxide.^38^^,^^39^

Alternatively, K_Ca_1.1 channel opening may lead to water efflux across the cell membrane and a reduction in TM and SC cell volume.^5^^,^^6^ Because aqueous humor passes through narrow tortuous spaces in the juxtacanalicular TM and through micron-sized pores in the inner wall endothelium of SC, relatively small changes in cell volume at flow-limiting sites can have significant effects on outflow facility. Consistent with this notion, the increase in outflow facility following perfusion with hypertonic saline coincides with a widening of open spaces in the juxtacanalicular TM.^40^ Similarly, treatment with nitric oxide decreases cell volume in TM and SC cells in vitro, and the time scale for cell volume change corresponds to the time scale for changing outflow facility.^6^^,^^15^ Blocking K_Ca_1.1 with IbTX inhibits both the effect of nitric oxide on TM and SC cell volume and on outflow facility.^6^^,^^13^^,^^15^ Thus K_Ca_1.1 channel opening appears to increase outflow facility by promoting cellular volume reduction and/or cell relaxation in the outflow pathway. Conversely, blocking K_Ca_1.1 with IbTX or MarTX should decrease outflow facility by inhibiting relaxation and/or cell volume decrease. In addition, because cell volume regulation is tightly coupled to mechanical stretch and contractility experienced by TM cells,^41^ it may not be possible to attribute the effects of K_Ca_1.1 to cell volume or contractility alone.

Other potassium ion channels, in addition to K_Ca_1.1, have similar effects on outflow facility. Opening the ATP-sensitive inward rectifier potassium channel 11 (K_ir_6.2) with cromakalim,^42^ or its prodrug CKLP1,^43^ increases outflow facility in human anterior segments and reduces IOP in wild-type mice, but not in homozygous null mice lacking K_ir_6.2.^42^ Despite the IOP-lowering effect of cromakalim/CKLP1, there was no detectible effect of CKLP1 on pressure-dependent outflow facility in either enucleated or in vivo mice,^43^ leading the authors to conclude that K_ir_ opening by CKLP1 may affect distal outflow.

Activity of K_Ca_1.1 is regulated by auxiliary *β*-subunits that affect channel sensitivity to intracellular calcium and membrane voltage.^16^^,^^17^ Of the four known *β*-subunits, we show that *β*_4_ is the most highly expressed in the anterior segment of the mouse eye. In cultured human SC cells, we show that *β*_4_ appears to be the sole *β*-subunit, and *β*_4_ is the primary *β*-subunit expressed by human TM cells. The *β*-subunit expression profile influences the pharmacology of the K_Ca_1.1 channel. IbTX and MarTX, for example, are venomous scorpion toxins that are evolutionarily selected to be highly specific for particular *β*-subunit combinations of K_Ca_1.1. IbTX completely blocks ion channels composed solely of pore-forming *α*-subunits that are present in all K_Ca_1.1 channels, but IbTX only partially blocks *β*_1_-containing K_Ca_1.1 channels (by ∼50%) and ineffectively blocks *β*_4_-containing K_Ca_1.1 channels (by ∼20%).^18^ MarTX, in contrast, is highly selective for *β*_4_-containing K_Ca_1.1 channels, with an IC50 value of 21 to 78 nM,^19^^,^^28^ and MarTX has a negligible effect on K_Ca_1.1 channels containing only *α-*subunits.^19^ MarTX also has a negligible effect on *β*_1_-containing K_Ca_1.1 channels for concentrations up to 400 nM with low cytoplasmic calcium.^44^ However, with high cytoplasmic calcium, MarTX enhances *β*_1_-containing K_Ca_1.1 channel activity with an EC_50_ of 495 nM.^45^ Taken together, the 100 nM concentration of MarTX used in this study should have had little effect on *β*_1_-containing K_Ca_1.1 channels, as *β*_1_ was the second most highly expressed *β*-subunit after *β*_4_ in TM cells and in mouse anterior segments.

In response to IbTX, we measured a significant decrease in outflow facility in enucleated mouse eyes. This suggests that at least some of the channels involved in facility regulation are IbTX-sensitive (i.e., *β*_4_-deficient), consistent with prior reports.^5^^,^^6^ However, MarTX, which selectively blocks *β*_4_-containing K_Ca_1.1 channels, had a nearly two-fold larger facility reduction relative to IbTX (∼35% vs. ∼16% average reduction). Although the MarTX and IbTX experiments were performed in separate cohorts such that the data are not directly comparable, the larger facility effect observed with MarTX suggests that the majority of K_Ca_1.1 channels involved in outflow facility regulation contain the *β*_4_-subunit. However, further study would be necessary to confirm within the same cohort whether MarTX truly has additive inhibitory effects beyond IbTX. Regardless, the magnitude of the facility reduction measured in response to MarTX was comparable to that previously reported in response to sphingosine 1-phosphate,^46^ reduced temperature,^47^ and prolonged exposure to dexamethasone.^48^ Thus *β*_4_-containing K_Ca_1.1 channels appear to be centrally important for the maintenance of outflow because blocking these channels significantly disrupts outflow function.

Using confocal microscopy, we localized expression of *β*_4_ to the juxtacanalicular TM, with punctate labeling observed along the endothelium of SC. This labeling pattern was consistent with *β*_4_ expression along cell processes that connect juxtacanalicular TM cells with inner wall cells.^32^^,^^33^^,^^48^^–^^50^ These processes experience significant biomechanical deformation in response to IOP elevation.^34^^,^^51^^–^^53^ As the juxtacanalicular TM and inner wall of SC are the primary sites of outflow resistance generation,^1^^,^^2^ this localization suggests a mechanosensory and regulatory role for *β*_4_. Specifically, we postulate the existence of a stretch-sensitive feedback loop whereby *β*_4_-containing K_Ca_1.1 channels are opened in response to IOP-induced stretch in the juxtacanalicular TM, triggering signaling pathways that lead to increased outflow facility to oppose the elevation in IOP. Previous studies have provided evidence that K_Ca_1.1 channels in bovine TM are directly sensitive to stretch, independent of cytosolic calcium levels.^12^ Even if K_Ca_1.1 channels are not directly stretch-activated, K_Ca_1.1 may be activated by elevated intracellular calcium that often occurs in response to stretch or other mechanical stimulation. Consequently, we hypothesize that the association with particular *β*-subunits influence the mechanosensitivity of K_Ca_1.1 in the juxtacanalicular TM. For example, the *β*_4_-subunit regulates shear and stretch-mediated mechanotransduction in intercalated cells of the kidney collecting duct.^54^^,^^55^ Similarly, *β*_4_ may influence mechanotransduction via K_Ca_1.1 in the juxtacanalicular TM.

Stretch induces secretion of several compounds from TM cells that increase outflow facility, such as adenosine triphosphate,^56^ metalloproteinases,^57^ and vascular endothelial growth factor.^58^ As K_Ca_1.1 appears to be involved in the stretch response in TM cells, it is possible that blocking K_Ca_1.1 may suppress stretch-induced release of these compounds to oppose the facility increase. K_Ca_1.1 is also involved in homeostatic regulation of cell volume, as occurs during the regulatory volume decrease following hypoosmotic shock. As regulatory volume decrease is associated with increasing outflow facility,^59^ blocking K_Ca_1.1 could potentially affect cell volume regulation in the TM to affect outflow facility.

## Conclusions

We have shown that *β*_4_ is the primary *β*-subunit of K_Ca_1.1 expressed in outflow pathway tissues. Blockade of *β*_4_-containing K_Ca_1.1 channels with MarTX leads to a physiologically significant decrease in outflow facility, which appeared to be larger than observed when blocking K_Ca_1.1 channels that lack *β*_4_ with IbTX. The *β*_4_-subunit is localized to the juxtacanalicular TM and inner wall of SC, where it may influence the response of these tissues to IOP-induced stretch or shear stress. The *β*_4_-subunit is thus important for outflow regulation, potentially by influencing mechanotransduction via K_Ca_1.1. Compounds that modulate *β*_4_-K_Ca_1.1 activity should therefore be explored as an approach to improve outflow and lower IOP for glaucoma therapy.

## Supplementary Material

Supplement 1

Supplement 2
