# Supplementary material for: The β4-Subunit of the Large-Conductance Potassium Ion Channel KCa1.1 Regulates Outflow Facility in Mice
Source: Invest Ophthalmol Vis Sci. 2020 Mar 23;61(3):41. doi: 10.1167/iovs.61.3.41 (PMC7401454; doi:10.1167/iovs.61.3.41)
Supplement: Supplement 1 [file iovs-61-3-41_s001.pdf]

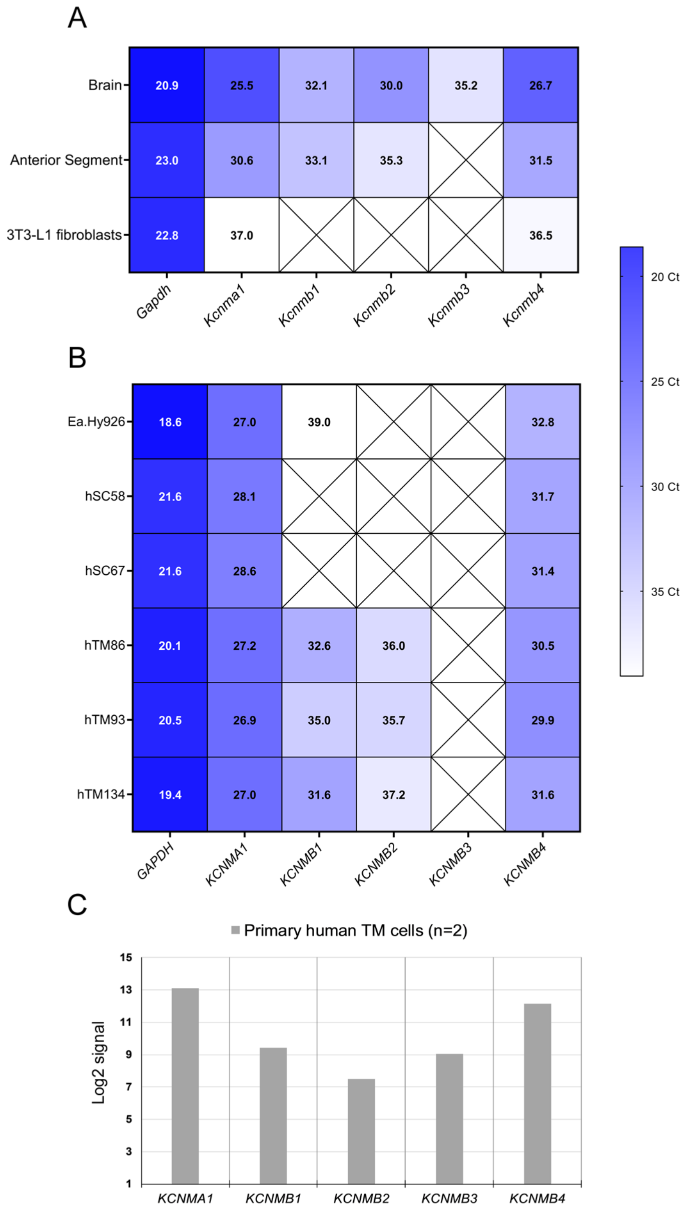

Supplemental Figure 1: A heatmap of mean cycle thresholds (Ct) for genes encoding the K_{Ca}1.1 channel α-subunit and the four β-subunits in mouse tissues (A) and human cells (B) measured by qPCR. The housekeeping gene, *GAPDH/Gapdh* is shown as a reference. Mouse tissues show mean Ct from three C57BL/6J mice. Crosses indicate samples that could not be amplified after 40 cycles. (C) Analysis of a previously published microarray data set [33] based on primary cultures of human TM cells (N=2, age: 30-60 years, post-mortem time <48 h) with no history of eye disease) from the North Carolina Eye Bank (NCEB) and National Disease Research Interchange (NDRI).
