# Supplementary material for: The β4-Subunit of the Large-Conductance Potassium Ion Channel KCa1.1 Regulates Outflow Facility in Mice
Source: Invest Ophthalmol Vis Sci. 2020 Mar 23;61(3):41. doi: 10.1167/iovs.61.3.41 (PMC7401454; doi:10.1167/iovs.61.3.41)
Supplement: Supplement 2 [file iovs-61-3-41_s002.pdf]

	TaqMan Assay ID	
**Gene**	**Human**	**Mouse**
KCNMA1/Kcnma1	Hs01119493_m1	Mm01268569_m1
KCNMB1/Kcnmb1	Hs00188073_m1	Mm00466621_m1
KCNMB2/Kcnmb2	Hs00175772_m1	Mm00511481_m1
KCNMB3/Kcnmb3	Hs03044885_m1	Mm01292438_m1
KCNMB4/Kcnmb4	Hs00929960_m1	Mm00465684_m1
GAPDH/Gapdh	Hs02758991_g1	Mm99999915_g1

Supplemental Table 1. TaqMan exon spanning primer/probe assays for detecting human and mouse gene expression of $K_{Ca}1.1$ channels *α* and *β*1-4. *GAPDH/Gapdh* was used as the housekeeping gene.
